# The Chaplain's Story

**Published:** 1918-12-28

**Authors:** 


### Miss Edith Cavell's Murder.

### THE CHAPLAIN'S STORY.

The Rev. H. S. T. Gahan, English chaplain in Brussels during its occupation by the Germans, relates in the *Daily Telegraph* how the news of the horrible crimes committed by the German Governor was the most awful shock the English community had to bear.

"We were a little band imprisoned here, and it was then the iron entered our soul, and we feel it still. Let me take you back to Christmas, 1914, and show you Miss Cavell as she was then. I went to her clinic, where she had a Christmas-tree and was giving a little Christmas party. The room was filled with people of all sorts and conditions. There were even some escaped British prisoners of war moving about quite freely—what risks we took in those days; we thought then the Germans were slow and careless, and we didn't know how deadly sure they were. I recall the scene distinctly, with Nurse Cavell standing in the midst, smiling and happy, with a kind word for everybody.

"One day, months afterwards, a boy came up to me in the street and said: 'The Germans have gone to Miss Cavell's clinic, sir; I saw their motor-car outside.' That was our first shock, though we knew nothing of what was going on, and only felt uneasy. Everything was done very secretly, and we heard nothing until a few days later, when, to my horror, someone came to me and said, 'The Germans have been there again, and have taken Miss Cavell away with them.' Then we heard she was in prison. There were all sorts of stories flying about, but we could find out nothing. Then, one evening after having been visiting in the country, I returned to my house and found on my desk a scribbled note: 'I want you to come to see me, to give the Communion to an Englishwoman who has not long to live.' It was written in English, but bore the signature of the German chaplain—a man of French extraction and named Lesure, of whom I must say that I found him a Christian gentleman.

"I went straight down to his lodgings. He came into the room looking very distressed, and said to me: 'I am very sorry to have to send for you. But you know Miss Cavell is in prison.' That made me feel queer, for I could not think what was coming next, and then he said: 'You know she is to be shot to-morrow morning?' I just sank back into a chair. Then he told me that with great difficulty he had got permission from the German military authorities for me to see her, and that I could go to her that evening. Then the German chaplain said: 'I asked Miss Cavell if she would like you to be with her at the execution, but she said, "No, I think it would be too much for him."' Of course, I would have gone, but how tender was that thought of her!

"It was then too late, the chaplain told me, for any change to be made by which I could be with her in her last moments, which were fixed for the following morning. So I left the chaplain's house and went direct to the prison at Saint Gilles. I went there expecting to find a woman distracted, a woman naturally terrified at the thought of her awful fate in a few hours, whom I would have to try to pull together, to control, and console. But when I saw Nurse Cavell face to face I found her perfectly calm and composed. She spoke naturally, in her customary quiet voice, and uttered no word that betrayed any trace in her of a feeling of animosity towards her judges. I have never seen anyone more perfectly sweet, composed, and quiet. It was just as if she were about to go to bed to get up the next morning and go about her ordinary life. The rest you know."

